# Comparison of hemostatic ability between spray coagulation and forced coagulation modes in endoscopic submucosal dissection in patients with early gastric neoplasms: a study protocol for multicenter randomized controlled trial (Spray-G trial)

**DOI:** 10.1186/s13063-023-07852-6

**Published:** 2024-01-15

**Authors:** Kosuke Maehara, Mitsuru Esaki, Yorinobu Sumida, Daisuke Yamaguchi, Kei Nishioka, Hitoshi Homma, Taisuke Inada, Kazuo Shiotsuki, Shin-Ichiro Fukuda, Hirotada Akiho, Tadahiro Nomura, Yumi Mizuta, Satoshi Ishida, Shun Fujimoto, Shunichiro Kimura, Yuichiro Tanaka, Kaori Hata, Noriko Shiga, Tsutomu Iwasa, Yusuke Kimura, Norimoto Nakamura, Yusuke Suzuki, Yosuke Minoda, Yoshitaka Hata, Haruei Ogino, Koshiro Tagawa, Eikichi Ihara, Yoshihiro Ogawa

**Affiliations:** 1 Department of Gastroenterology, Kitakyushu Municipal Medical Center, 2-1-1 Bashaku, Kokurakita-Ku, Kitakyushu, Fukuoka Japan; 2 Department of Medicine and Bioregulatory Science, Graduate School of Medical Sciences, Kyushu University, 3-1-1 Maidashi, Higashi-Ku, Fukuoka Japan; 3 Department of Gastroenterology, Harasanshin Hospital, 1-8, Taihaku-Cho, Hakata-Ku, Fukuoka Japan; 4 Department of Gastroenterology, National Hospital Organization Ureshino Medical Center, Ureshino, Japan; 5 Department of Gastroenterology, Saiseikai Futsukaichi Hospital, 3-13-1 Yumachi, Chikushino, Fukuoka Japan; 6 Department of Gastroenterology, Fukuoka Central Hospital, 2-6-11 Yakuin, Chuo-Ku, Fukuoka Japan; 7 Department of Gastroenterology and Metabolism, Graduate School of Medical Sciences, Kyushu University, 3-1-1 Maidashi, Higashi-Ku, Fukuoka Japan; 8 Center for Clinical and Translational Research, Kyushu University Hospital, 3-1-1 Maidashi, Higashi-Ku, Fukuoka Japan

**Keywords:** Endoscopic submucosal dissection, Gastric neoplasms, Spray coagulation mode, Hemostasis

## Abstract

**Background:**

Endoscopic submucosal dissection (ESD) is the standard treatment for early gastric neoplasms (EGN). Controlling intraoperative bleeding is crucial for ensuring safe and reliable procedures. ESD using the spray coagulation mode (SCM-ESD) has been developed to control bleeding more effectively than ESD using the conventional forced coagulation mode (FCM-ESD). This study aims to compare the hemostatic efficacies of SCM-ESD and FCM-ESD.

**Methods:**

This multicenter, prospective, parallel, randomized, open-label superiority trial will be conducted in five Japanese institutions. Patients with a preoperative diagnosis of intramucosal EGC will be randomized to undergo either SCM-ESD or FCM-ESD. The primary outcome measure is the completion of ESD with an electrosurgical knife alone, without the use of hemostatic forceps. Secondary outcomes include the number and duration of hemostasis using hemostatic forceps, procedure time, curability, and safety. A total of 130 patients will be enrolled in this study.

**Discussion:**

This trial will provide evidence on the hemostatic efficacy of SCM-ESD compared with FCM-ESD in patients with intramucosal EGN, potentially improving the safety and reliability of ESD procedures.

**Trial registration:**

The trial has been registered at the University Hospital Medical Information Network Clinical Trials Registration (UMIN-CTR) as UMIN000040518. The reception number is R000054009.

**Supplementary Information:**

The online version contains supplementary material available at 10.1186/s13063-023-07852-6.

## Introduction

### Background and rationale {6a}

Endoscopic resection (ER) is the standard treatment for early gastric neoplasms (EGN) with a negligible risk of lymph node metastasis [1]. ER is a minimally invasive treatment that preserves organ function, leading to a better post-procedure quality of life than surgery [2]. Endoscopic mucosal resection (EMR), the first form of ER, was developed to treat EGN. However, snaring techniques have limitations, particularly in terms of the piecemeal resection of large or ulcerated lesions, leading to difficulties in accurate histological assessment and a high risk of local recurrence. Endoscopic submucosal dissection (ESD) using an electrosurgical knife has been developed to overcome these limitations. ESD allows en bloc resection of large or ulcerated lesions, resulting in accurate histological assessment and a reduced risk of local recurrence [3, 4]. Despite its higher curative potential, ESD is reported to be more difficult to perform, with a longer procedure time and higher adverse event rates, including bleeding and perforation, than EMR [3, 4]. Controlling intraoperative bleeding during ESD is crucial for a safe and reliable procedure [5]. In cases of intraoperative bleeding or exposed vessels, the coagulation wave of the electrosurgical knife is used to cauterize the area. In more severe cases, hemostatic forceps may be employed if it is difficult to control bleeding or vessels with a knife alone [6].

In ESD, two basic electrocautery patterns of an electrosurgical unit are employed: cut current and coagulation current [7]. Cut current is mainly used for mucosal incisions, whereas coagulation current is used for submucosal dissection and hemostasis. Forced coagulation mode (FCM) is conventionally used as the coagulation current [8, 9]. VIO3 (ERBE, Germany) is the latest high frequency unit (HFU), which has been developed to improve the performance of the electrosurgical knife in ESD. VIO3 facilitates submucosal dissection via coagulation currents. The spray coagulation mode (SCM), with a higher peak voltage and shorter duty cycle, possessed greater coagulation ability than conventional FCM [10–13]. Recently, ESD with SCM in VIO3 (SCM-ESD) has been developed to control procedure-related bleeding more effectively than FCM (FCM-ESD). Our pilot data showed that SCM-ESD reduced the use of hemostatic forceps as a rescue device by 28% while maintaining high curability and safety. To confirm the hemostatic efficacy of SCM-ESD, we aim to conduct a multicenter randomized controlled trial to compare the clinical outcomes of SCM-ESD and FCM-ESD.

### Objectives {7}

This study aims to investigate the hemostatic efficacy of SCM-ESD and FCM-ESD in patients with EGN.

### Trial design {8}

**Fig. 1:**
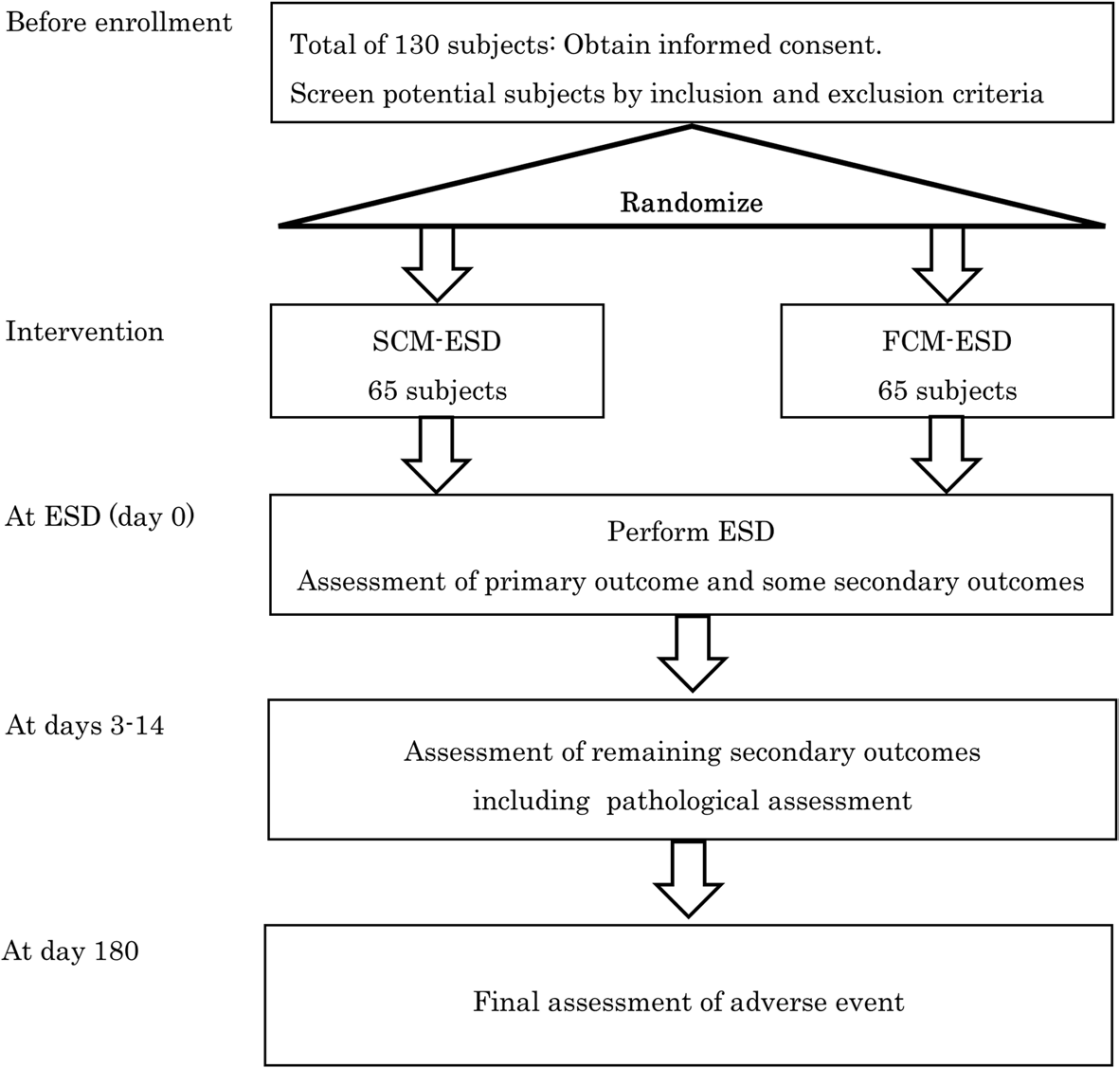
Schematic of the study design. SCM-ESD, spray coagulation mode endoscopic submucosal dissection; FCM-ESD, forced coagulation mode endoscopic submucosal dissection; ESD, endoscopic submucosal dissection

This is a prospective, parallel, randomized, open-label superiority trial. The trial design adheres to the recommendations of the Standard Protocol Items: Recommendations for Interventional Trials (SPIRIT) checklist (Additional file 2) [ [14]. Patients with a preoperative diagnosis of EGN are enrolled and randomly assigned to one of two interventional arms: SCM-ESD or FCM-ESD. A flowchart of the trial design is illustrated in Fig. 1.

## Methods: participants, interventions and outcomes

### Study setting {9}

This multicenter trial will be conducted across five institutions in Japan: Kitakyushu Municipal Medical Center, Kyushu University Hospital, Saiseikai Futsukaichi Hospital, Fukuoka Central Hospital, and the National Hospital Organization Ureshino Medical Center.

### Eligibility criteria {10}

The eligibility criteria for this study include the following: (i) patients with lesions endoscopically diagnosed as EGN and eligible for ESD, (ii) patients with lesions diagnosed by endoscopic biopsy as gastric adenomas or adenocarcinomas, (iii) patients with age ≥ 20 years at the time of consent, (iv) patients with Eastern Cooperative Oncology Group Performance Status of 0–2, and (vi) patients capable of understanding the study explanations and providing signed consent. Exclusion criteria for this study include the following: (i) patients with a history of gastric surgery, (ii) patients currently undergoing dialysis, (iii) patients requiring perioperative heparin administration, (iv) patients with contraindications to endoscopy, and (v) patients deemed inappropriate by the investigators for the study.

### Who will take informed consent? {26a}

The investigator will thoroughly explain the details of the trial to the potential patients, including the benefits and risks associated with the two treatment procedures. If patients express a willingness to participate, written informed consent for the trial will be obtained from the patients.

### Additional consent provisions for collection and use of participant data and biological specimens {26b}

If the patients agree to participate, additional written informed consent will be obtained to collect biological samples for histopathological assessment. The potential for secondary use of the samples and information obtained from this trial will be explained to the participants. These samples will be stored in a freezer in a locked laboratory for at least 5 years after the completion of the trial and then properly disposed of in accordance with the Kyushu University Standard Operating Procedures for the Storage of Samples and Information Obtained from Human Subjects.

## Interventions

### Explanation for the choice of comparators {6b}

The enrolled patients will be randomized to receive either SCM-ESD or FCM-ESD. FCM-ESD was selected as the control comparator because FCM is conventionally used as the coagulation current during ESD.

### Intervention description {11a}

As for operators, this trial will be conducted at institutions where ESD for EGN is performed regularly. Experienced endoscopists specialized in endoscopic diagnosis and treatment will exclusively perform ESD procedures in this trial. The criteria for skilled endoscopists are as follows: (i) completion of the postgraduate clinical training system in Japan for more than 2 years and involvement in endoscopic diagnosis and treatment, (ii) experience with at least1000 cases of endoscopy, and (iii) experience with more than 30 cases of ESD. In principle, a single operator will be responsible for completing the ESD procedure. However, a temporary or permanent operator change to a more skilled supervisor will be allowed in the following cases, prioritizing patient safety: (i) prolonged procedure time of ≥ 60 min for ESD, (ii) one instance of hemostasis requiring ≥ 10 min, (iii) occurrence of intraoperative perforation, or (iv) cases in which the supervisor deems the operator change necessary. Any temporary or permanent changes in the operators will be recorded.

As for equipment and setting, ESD will be performed using upper gastrointestinal therapeutic endoscopes (GIF-Q260J and GIF-H290T; Olympus, Tokyo, Japan) equipped with disposable hoods (not regulated). An injection needle (not regulated) will be used for submucosal injection with hyaluronic sodium or alginate sodium as the injection solution. ProKnife (Boston Scientific, Tokyo, Japan) will be utilized for various aspects of both SCM-ESD and FCM-ESD procedures including marking, mucosal incision, submucosal dissection, and hemostasis [15, 16]. HemoStat-Y (Pentax, Tokyo, Japan) will be used as the hemostatic forceps. The HFU used in this trial will be VIO3. The following settings will be employed for the electrosurgical knife on the HFU: during incision, end-cut I mode with effect 1 (ranging 1–3), duration 2 (ranging 1–3), and interval 1 (ranging 1–3); during submucosal dissection and hemostasis in FCM-ESD, forced coagulation mode with effect 5 (ranging 4–6); during submucosal dissection and hemostasis in SCM-ESD, spray coagulation mode with effect 5 (ranging 4–7). The setting of the HFU for the hemostatic forceps will be the bipolar-soft coagulation mode with Effect 5 (ranging 4–6) with the quick-start mode activated.

As for ESD procedure, in principle, ESD will be performed for en bloc resection of a target lesion using an electrosurgical knife. However, if en bloc resection is not feasible owing to certain circumstances, alternative strategies such as piecemeal resection or additional ablation techniques such as argon plasma coagulation or hot biopsy may be employed to prevent residual tissue. After identifying the lesion, circumferential marking dots will be made placed approximately 2–3 mm outside the lesion using the tip of the knife. An injection needle will then be introduced from outside the markings, and a viscous solution will be injected into the submucosal layer beneath the lesion. After confirming elevation of the lesion, an initial mucosal incision will be made outside the marking using a knife. After completing the circumferential mucosal incision, submucosal dissection will be initiated using a knife. Traction assistance can be provided in the direction of the operator. Additional local injections can be administered using either the injection needle or the tip of the knife as the mucosal incision or submucosal dissection progresses. The volume of solution injected from the injection needle will be recorded. Dissection will continue until the lesion is excised with a knife. In cases of bleeding during mucosal incision or submucosal dissection, initial hemostasis will be attempted by coagulation with the tip of a knife. However, if bleeding cannot be controlled using a knife alone, hemostatic forceps will be employed as a rescue device. Forceps will be used to grasp the bleeding vessels, followed by coagulation. The criteria for transitioning to hemostatic forceps are as follows: (i) complete hemostasis cannot be achieved within 30 s using the knife; (ii) if the operator determines that bleeding is difficult to control with the knife alone; or (iii) if the operator determines that dealing with the exposed vessel using the knife alone is challenging. Transitioning to hemostatic forceps will be permitted if (ii) or (iii) is met, even if (i) is not fulfilled, to ensure patient safety. If hemostatic forceps are employed, hemostasis with the forceps will continue until the bleeding is completely stopped. The number and duration of hemostatic forceps used will be also recorded. The use of other devices that are not regulated may be allowed based on the operator’s judgment and such instances will be recorded.

As for pathological assessment, the specimens will be fixed on a plastic plate and sliced at 2-mm intervals. Pathological diagnoses will be made by pathologists at each participating institution following the Japanese Classification of Gastric Carcinoma [17].

### Criteria for discontinuing or modifying allocated interventions {11b}

If hemostasis with hemostatic forceps is required more than five times during SCM-ESD or FCM-ESD, transitioning from SCM-ESD to FCM-ESD or from FCM-ESD to SCM-ESD will be allowed for safety reasons. Such a conversion will not constitute a protocol deviation that will be duly recorded.

### Strategies to improve adherence to interventions {11c}

No specific strategies have been established to improve patient adherence to interventions because the focus of this trial is primarily on the ESD procedure.

### Relevant concomitant care permitted or prohibited during the trial {11d}

To prevent delayed bleeding, proton pump inhibitors or potassium-competitive acid blockers can be administered daily, starting from the date of ESD and continuing until discharge.

The concomitant use of antithrombotic agents, excluding continuous heparin, will be permitted throughout the study period, following the Japanese guidelines for gastroenterological endoscopy in patients undergoing antithrombotic treatment [18].

After treatment, patients will be kept on a fasting regimen and administered an intravenous drip. Oral intake will be resumed 2–3 days after ESD, starting with a liquid or soft diet, and gradually transitioning to a normal diet.

### Provisions for post-trial care {30}

Proton pump inhibitors or potassium-competitive acid blockers can be administered to patients for up to eight weeks from the date of ESD to prevent delayed bleeding.

No special compensation will be provided, because it is not anticipated that any harm will result from participating in this study.

### Outcomes {12}

The primary outcome is successful completion of ESD using an electrosurgical knife alone, which is considered an important indicator reflecting the hemostatic ability to control intraoperative bleeding. If hemostatic forceps are required as a rescue device to achieve hemostasis prior to tumor retrieval, the procedure will be considered a failure. Secondary outcome includes the number and duration of hemostasis performed with hemostatic forceps; procedure time, including total ESD time, mucosal incision time, and submucosal dissection time; speed of submucosal dissection; en bloc resection rate; complete resection rate; curative resection, evaluated based on endoscopic curability (A or B); degree of each endoscopic curability category; thickness of the submucosal layer in the resected specimen; type and volume of submucosal injective solution used; occurrence of operator change; and adverse events. Total ESD time is divided into mucosal incision time and submucosal dissection time. The continuous outcome data will be analyzed without any categorization.

Outcome without histopathological assessments will be evaluated at ESD. Outcomes with histopathological assessments including complete resection rate; curative resection, evaluated based on endoscopic curability (A or B); degree of each endoscopic curability category; and thickness of the submucosal layer in the resected specimen will be evaluated within 1 week after ESD.

### Participant timeline {13}

**Fig. 2:**
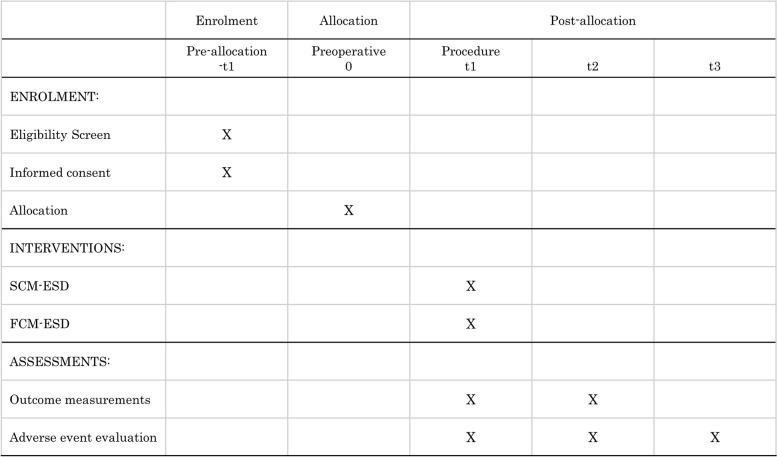
SPIRIT flow diagram. SCM-ESD, spray coagulation mode endoscopic submucosal dissection; FCM-ESD, forced coagulation mode endoscopic submucosal dissection

The participants’ timelines are shown in Fig. 2. The protocol adheres to the Standard Protocol Items: Recommendations for Interventional Trials (SPIRIT) guidelines.

### Sample size {14}

In our previous pilot study comparing the outcomes of SCM-ESD and FCM-ESD for the same subjects as in this study, the completion rate of ESD with the electrosurgical knife alone in SCM-ESD was 62.5% (40/64) compared to 34.6% (9/26) in FCM-ESD, resulting in a difference of 27.9%. Considering the variability in ESD outcomes among institutions, we assumed a 25% additive effect of SCM-ESD over FCM-ESD on the completion rate. The required number of cases was determined using the *χ*^2^ test with a two-sided alpha significance level of 5% and a power of 80%, resulting in a total required number of 62 case per group (124 cases in total). Considering a dropout rate of approximately 5% for ineligible patients, we calculated that 65 patients per group (130 patients in total) would be required.

### Recruitment {15}

EGN is detected by upper gastrointestinal endoscopy performed at either the referral or participating institutions involved in this study. Patients are required to visit the outpatient clinic for an explanation of ESD prior to treatment. The investigators then review the eligibility and exclusion criteria for all potential patients. The recruitment period has been designed for two years. The number of eligible patients at all institutions per month is estimated to be approximately 20. Assuming a 30% consent rate, enrollment is expected to be completed in 2 years.

## Assignment of intervention: allocation

### Sequence generation {16a}

Upon obtaining patient consent, the investigator will register the patient in the database of the UMIN Medical Research Support Cloud version, UMIN INDICE Cloud, which serves as a web-based central randomization system. Each patient will be assigned a unique identification number for registration. The registration process will only be accepted if all the required data are provided. After confirming the eligibility on the registration screen, a registration number will be generated. The UMIN INDICE cloud will facilitate both immediate and concealed allocations. Registered patients will be randomized (1:1) into either the SCM-ESD or FCM-ESD groups using dynamic balancing, employing a minimization method based on tumor location (upper or middle third of the stomach vs. lower third of the stomach), tumor size (0–20 mm vs. > 20 mm), and the use of thrombotic agents (presence vs. absence).

### Concealment mechanism {16b}

A web-based central randomization system with a validated password will ensure concealment of the randomization sequence.

### Implementation {16c}

A web-based central randomization system will generate randomization using an allocation sequence. One investigator will oversee the randomization system, but will not participate in patient enrollment or study treatment.

## Assignment of interventions: blinding

### Who will blinded {17a}

Neither the patients nor investigators will be blinded to the allocated treatment.

### Procedure for unblinding if needed {17b}

Not applicable, as this study is unblinded.

## Data collection and management

### Plans for assessment and collection of outcomes {18a}

The investigator will gather data, including general information, eligibility criteria, and exclusion, at the time of registration. Perioperative and postoperative data will be inputted by the investigator, referencing medical records as appropriate. All registration and outcome information will be stored in the UMIN INDICE Cloud.

### Plans to promote participant retention and complete follow-up {18b}

Upon registration, all assessments of study outcomes and follow-up will be conducted during the hospital stay. Furthermore, follow-up on procedure-related adverse events will be continued for six months after treatment. The principal investigator will continuously monitor the retention rate.

### Data management {19}

Each investigator will register and input data into the UMIN INDICE cloud. The investigators must ensure that the data are accurate and complete. The principal investigator will confirm data adequacy. The data are stored in the UMIN INDICE cloud and accessible only to research personnel trained in confidentiality and privacy.

### Confidentiality {27}

All data stored in the UMIN INDICE cloud will be protected from access by third parties by setting identification numbers and passwords, encryption using 128 bits SSL and VPN, double firewalls, and a monitoring system for unauthorized access and intrusion. No information that can easily identify the individuals is stored in the dataset. The correspondence sheet linking the patient and identification number will be stored in a lockable box. All information retrieved from the cloud will be stored on a computer with a password set for 10 years after the completion of the trial.

### Plans for collection, laboratory evaluation, and storage of biological specimens for genetic or molecular analysis in this trial/future use {33}

Investigators will obtain informed consent from the patients to collect biological samples for histopathological assessment. These samples will be securely stored in a freezer within a locked laboratory for at least five years following the completion of the study and then properly disposed of in accordance with each institution’s Standard Operating Procedures for the Storage of Samples and Information Obtained from Human Subjects. Any secondary use of the samples and information in future trials will be conducted only after obtaining written consent from the participants and receiving approval from the Institutional Review Committee for the new trial protocol.

## Statistical methods

### Statistical methods for primary and secondary outcomes {20a}

With regard to the primary outcome as the completion of ESD with the knife alone, the two groups will be compared using the Cochran-Mantel–Haenszel test stratified by lesion location (upper or middle third of the stomach vs. lower third of the stomach), lesion size (0–20 mm vs. 21 mm or more), and presence of antithrombotic agents (continued, discontinued, or not administered). If SCM-ESD significantly outperforms FCM-ESD (two-sided significance level of 5%), we will conclude that SCM-ESD is a more useful treatment method than FCM-ESD.

With regard to secondary outcomes, the number and duration of hemostasis using hemostatic forceps and procedure time will be analyzed using the Wilcoxon rank-sum test. The speed of dissection, thickness of the submucosal layer in the resected specimen, and volume of the submucosal injection solution will be analyzed using *t*-tests. En bloc resection, complete resection, curative resection, severe thermal damage to the resected specimen, type of submucosal injection solution, operator change, and occurrence of adverse events will be analyzed using Fisher’s exact test.

### Interim analyses {21b}

No interim analyses are planned. Considering the high curability and safety of FCM-ESD and SCM-ESD reported in the previous studies and our pilot study, patients would not be seriously disadvantaged by completing the study without an interim analysis [19].

### Methods for additional analyses (e.g., subgroup analyses) {20b}

As for subgroup analysis, primary outcomes and some secondary outcomes, including the number and time of hemostasis with hemostatic forceps and total procedure time, will be analyzed according to the tumor location, tumor size, antithrombotic agent use, experience of ESD, and pathological ulceration.

### Method in analysis to handle protocol non-adherence and any statistical methods to handle missing data {20c}

The analysis will be performed primarily on the largest population of enrolled patients, excluding those who do not receive trial treatment, those with serious ethical guideline violations, and those with missing primary outcome data. In principle, missing data will not be imputed because it is assumed that there will be quite few missing data due to the design of this trial.

### Plans to give access to the full protocol, participant-level data and statistical code {31c}

The datasets analyzed in this trial, statistical codes, and full protocol will be available from the corresponding author upon reasonable request.

## Oversight and monitoring

### Composition of the coordinating center and trial steering committee {5d}

The coordinating center is comprised of experts in gastroenterology and endoscopy. They are responsible for overseeing the trial and managing the protocol and trial-related documents. The trial steering committee is composed of members, including principal investigators from each institution. They are responsible for the overall management of the trial and implementation of the protocol at each institution. Meetings will be held once a month to discuss protocol compliance and any changes to the protocol.

### Composition of the data monitoring committee, its role and reporting structure {21a}

Members of the data monitoring committee are responsible for verifying the progress of the trial and ensuring that it is conducted, recorded, and reported in accordance with relevant laws, guidelines, and study protocols. The committee members are independent of the clinical trial stakeholders and are not involved in patient registration or treatment.

### Adverse event reporting and harms {22}

When an adverse event is recognized, the investigator must promptly take appropriate measures and document the event in the medical records or other relevant documents. If the trial treatment is discontinued or if treatment for an adverse event is required, the patients must be informed accordingly.

The reporting procedures are as follows: (i) in the event of a serious adverse event, the investigators must take necessary measures including explaining to the patient and promptly report it to the principal investigator, following the “Procedure Manual for Handling Safety Information in Human Medical Studies”; (ii) when being aware of a serious adverse event, the principal investigator should promptly take necessary measures, ensure appropriate responses, and create a “Serious Adverse Event Report,” and submit it to the hospital director through the secretariat of the Clinical Trial Ethics Revie Committee. Furthermore, the principal investigator should promptly share information related to the occurrence of adverse events with other trial investigators.

### Frequency and plans for auditing trial conduct {23}

The project management group will meet once a month to review the trial. The meeting will include a review of registration, consent procedures, protocol adherence, adverse events, and quality of control of all data. The trial steering group and the independent data monitoring and ethics committee will meet to review conduct throughout the trial period once per 6 months.

### Plans for communicating important protocol amendments to relevant parties (e.g., trial participants, ethical committees) {25}

If any protocol modifications are required, they are submitted to the IRB for approval prior to implementation. A revised copy will be stored, and the protocol in the clinical trial registry will be updated. This study is an investigator-initiated clinical trial with no trial sponsor. The principal investigator will be communicated to all study personnel including investigators in each institution on time. Participants will also be informed orally or in writing of any amendments to the protocol.

### Dissemination plans {31a}

The findings of this trial will be presented at domestic and international conferences and disseminated through the publication of papers in peer-reviewed journals. No personally identifiable information will be included during this process.

## Discussion

This trial aims to provide evidence supporting the superiority of hemostatic ability in SCM-ESD compared with FCM-ESD for patients with intramucosal EGN. Completion of ESD with the knife alone, without the use of hemostatic forceps as the primary outcome measure, can be achieved when bleeding control using the knife is sufficient during ESD. The higher completion rate in SCM-ESD indicates that the bleeding control ability using the knife is superior to that of conventional FCM-ESD. The number and duration of hemostasis with hemostatic forceps, as secondary outcomes, also reflect the hemostatic ability of the knife during ESD. If bleeding control with the knife is better, the number and duration of hemostasis with hemostatic forceps can be expected to decrease, even when needed. Additionally, other secondary outcome measures, such as procedure time, en bloc and complete resection rates, and adverse events, will provide a comprehensive understanding of the potential advantages of SCM-ESD over FCM-ESD.

In conclusion, the findings of this multicenter randomized controlled trial are expected to provide valuable evidence on the hemostatic efficacy of SCM-ESD compared with FCM-ESD in patients with intramucosal EGN. These results could lead to the adoption of SCM-ESD as the preferred treatment method for EGN, potentially improving the safety and reliability of ESD procedures.

## Trial status

Recruitment for this RCT began April 4, 2022, and the first participant was enrolled on April 5, 2022. We initially planned for recruitment to end on March 31, 2024 (24 months). However, owing to the rapid pace of case accumulation, recruitment was completed ahead of schedule on February 21, 2023. Follow-up on procedure-related adverse events is continued. Study protocol data: initial approval, March 23, 2022; current version approval, August 25, 2022.

### Supplementary Information

**Additional file 1.**

**Additional file 2.**

## Data Availability

The final datasets will be available from the corresponding author upon reasonable request after completion of the study.

## Abbreviations

ESD: Endoscopic submucosal dissection
EGN: Early gastric neoplasms
SCM: Spray coagulation mode
FCM: Forced coagulation mode
SCM-ESD: Spray coagulation mode endoscopic submucosal dissection
FCM-ESD: Forced coagulation mode endoscopic submucosal dissection
ER: Endoscopic resection
EMR: Endoscopic mucosal resection
HFU: High frequency unit
SPIRIT Standard protocol Items: Recommendations for Interventional Trials
UMIN-CTR: University Hospital Medical Information Network Clinical Trials Registration
IRB: Institutional Review Board
UMIN: University Hospital Medical Information Network
INDICE: Integrated Network of Clinical Research Information and Electronic Medical Records
RCT: Randomized controlled trial
SAE: Serious adverse event
