# Supplementary material for: Comparison of hemostatic ability between spray coagulation and forced coagulation modes in endoscopic submucosal dissection in patients with early gastric neoplasms: a study protocol for multicenter randomized controlled trial (Spray-G trial)
Source: Trials. 2024 Jan 15;25:53. doi: 10.1186/s13063-023-07852-6 (PMC10788983; doi:10.1186/s13063-023-07852-6)
Supplement: Supplementary file 1 — Additional file 1. [file 13063_2023_7852_MOESM1_ESM.pdf]

臨床試験のご説明

臨床試験課題名：胃上皮性腫瘍に対する<br>従来法ESDおよびSpray-ESDの無作為比較試験

研究代表機関
北九州市立医療センター・消化器内科
研究代表者　隅田　頼信

北九州市立医療センター版
版番号　１．０版
作成年月日　2022年2月28日
（共通版　１．０版　2022年2月28日作成）

臨床試験のご説明

臨床試験課題名： 胃上皮性腫瘍に対する従来法ESDおよびSpray-ESDの無作為比較試験

はじめに

この説明文書は、あなたに、「胃上皮性腫瘍に対する従来法ESDおよびSpray-ESDの無作為比較試験」の内容を正しく理解していただき、あなたの自由な意思にもとづいて、この臨床試験に参加するかどうかを判断していただくためのものです。この説明文書をお読みになり、担当医からの説明を聞かれた後、十分に考えてからこの試験に参加するかどうかを決めて下さい。たとえ参加されなくても、今後の治療に不利益になることはありません。また、不明な点があればどんなことでも気軽に質問して下さい。なお、ご参加いただける場合は、別紙の「同意文書」にご署名のうえ、担当医師にお渡しください。

1. 臨床試験について

私達は最新の治療を患者さんに提供するために、病気の特性を試験し、診断法、治療法の改善に努めています。このような診断や治療の有効性や安全性の検討等を行うことを一般に「臨床試験」と言います。この臨床試験は、地方独立行政法人北九州市立病院機構　治験・臨床研究審査委員会で審議された上で、当院の病院長の許可を受けて実施されます。

2. あなたの病気について

あなたは、胃に発生する腫瘍のうち上皮性腫瘍（胃腺腫もしくは胃腺癌)といわれる診断がついています。この病気は、胃の粘膜層から発生する腫瘍で、将来悪性転化する可能性がある疾患もしくは早期の悪性疾患の状態と考えられています。癌化し、無治療で経過観察した場合には、リンパ節や他臓器への転移の危険性が出てきます。胃から離れたリンパ節や多臓器への転移を来した場合にはステージ4の状態となり、根治および救命することが極めて困難となります。そのため、早期発見および早期治療が必要となります。あなたの病気は、悪性転化する前の良性の状態もしくは、悪性ですが極めて早期の状態です。
(あなたの病気、及び病気の進み具合など、より詳細については担当医から説明があります)

3. あなたの病気に対する治療法について

胃腺腫および胃腺癌を根治するためには、局所切除が必要となります。以前は、外科的に開腹もしくは腹腔鏡で胃の周りのリンパ節を含めた胃切除が行われていました。しかし、内視鏡技術の普及に伴い、胃腺腫およびリンパ節転移のない胃腺癌に対して内視鏡治療が普及してきました[1,2]。
内視鏡治療導入当初は、スネアという輪のような器具を用いて、腫瘍を絞扼して電流を流して切除する内視鏡的粘膜切除術(endoscopic mucosal resection: EMR)が行われていました。その後、より治癒の可能性が高い内視鏡治療方法として高周波ナイフを用いた内視鏡的粘膜

下層剥離術(endoscopic submucosal dissection: ESD)が開発されました[3]。この方法の開発により、大きな病変や直下が瘢痕化して固くなっている病変でも切除できるようになりました[4]。一括切除を高める目的で、ほとんどの症例で ESD が選択されています。しかし高周波ナイフ（電気メス）により病変を剥離していくESDの手技は難易度が高く、EMRより治療時間が長く、合併症のリスクが高いことが報告されています。その中で、ESD の術中出血をコントロールすることは、治療の進行に大きく影響します。通常、術中に出血を来した場合や血管が露出した場合には、ナイフによる焼灼止血を行います。しかし、ナイフで出血のコントロールが困難な場合には、止血専用の鉗子デバイスで救済的に止血を行います。

従来のナイフ（電気メス）の設定として、主に Forced 凝固が用いられてきました。しかし、その設定ではナイフでの出血コントロールが困難となり、止血鉗子による救済を用いる場面が多くみられていました。そこで最近では、より凝固能(止血)が高い Spray 凝固の設定で、止血力の高い切開を行うことで、ナイフによる出血コントロールが改善するのではないかと考えられるようになりました。我々の過去の検討では、Spray 凝固を用いた ESD(Spray-ESD)は従来の方法より止血鉗子による救済を行う症例を約 28%減らすことができました。しかし、科学的に平等な 2 条件で従来法 ESD と Spray-ESD の治療成績を比較した報告がないため、どちらがより有効な治療法であるかの結論が得られていません。

[1] Tada M, Murakami A, Karita M, et al. Endoscopic resection of early gastric cancer. Endoscopy 1993; 25: 445-450

[2] Libanio D, Braga V, Ferraz S, et al. Prospective comparative study of endoscopic submucosal dissection and gastrectomy for early gastric neoplastic lesions including patients' prospectives.

[3] Ono H, et al: Endoscopic mucosal resection for treatment of early gastric cancer. Gut 2001; 48(2): 225-229.

[4] Park YM, Cho E, Kang HY, et al. The effectiveness and safety of endoscopic submucosal dissection compared with endoscopic mucosal resection for early gastric cancer: a systematic review and metaanalysis. Surg Endosc 2011; 25: 2666-2677.

従来の ESD と Spray-ESD の手技の説明は下記に示します(図 1)。

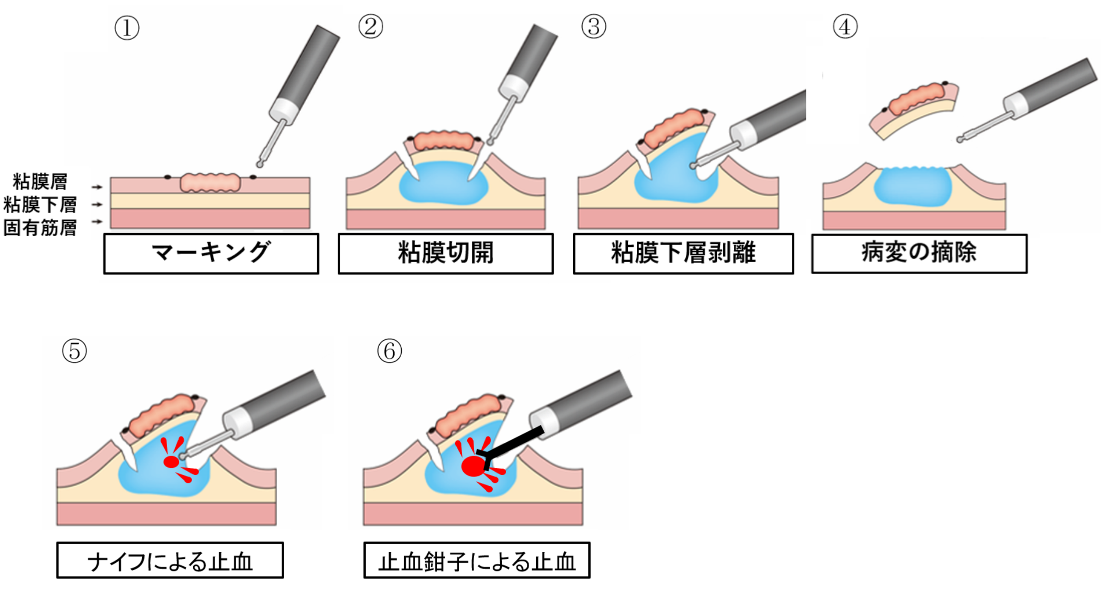

図 1　従来法 ESD および Spray-ESD の模式図
　① マーキング ②粘膜切開 ③粘膜下層剥離 ④病変の摘除
　⑤ナイフによる止血 ⑥止血鉗子による止血

従来法 ESD および Spray-ESD の共通手技:
胃カメラを挿入し、病変の観察を行います。内視鏡の内部にある穴から高周波ナイフの先端を出します。①病変の範囲を確認し、周囲に目印となる焼き印(マーキング)を付けます。②その後、病変の下に粘性のある液体を注入します。病変が膨隆したことを確認し、マーキング外側の粘膜(第 1 層)を切開します。③粘膜の切開に引き続いて、病変直下の粘膜下層(第 2 層)を剥離していきます。④剥離を進めていき、病変を摘除します。
　粘膜下層には血管が豊富に存在し、粘膜切開および粘膜下層剥離の途中に出血を来す場合があります。穿孔に注意し、出血時には止血を行いながら手技を進めていきます。初期の止血方法として、ナイフの凝固波を用いて止血を試みます。しかし、ナイフ単独での止血が困難な場合には救済方法として止血鉗子による止血方法に切り替えます。

　従来法 ESD
　従来法 ESD での、先端系ナイフを用いた凝固波の設定は Forced 凝固モード effect 5 (4-6)とします。ESD 中、術者の判断で必要に応じて設定を調節していきます。

　Spray-ESD
　Spray-ESD 群での、先端系ナイフを用いた凝固波の設定は Spray 凝固モード effect5(4-6)とします。ESD 中、術者の判断で必要に応じて設定を調節は可能します。

4. この臨床試験の目的、背景、意義

臨床研究の目的は、胃上皮性腫瘍と診断された患者様に対する内視鏡治療法として、新しく開発した Spray-ESD が、従来法 ESD と比較して、出血コントロールが難しくなり止血鉗子による救済を行う割合を減らすことができるかを検討することです。この研究の結果は、今後、胃上皮性腫瘍と診断患者さんに対する内視鏡治療法を考「える上で非常に重要なものとなります。

5.この臨床試験で使用する薬剤・医療機器について

使用する医療機器の概要

① 試験機器:高周波ナイフ:　ProKnife (Boston Scientific; 図 2)

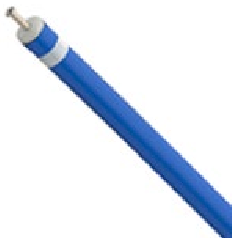

図 2 ProKnife(先端が出た状態)

使用方法

ESD のマーキング、粘膜切開および粘膜下層剥離を行う先端系ナイフです。

先端が出た状態で固定します(図 2)。この状態で、マーキング、粘膜切開および粘膜下層剥離を行います。出血時には凝固波で止血を行います。また剥離途中に先端部から追加局注を行います。

① 止血鉗子：Hemostat-Y (Pentax)

使用方法

先端系ナイフで出血コントロールがつかない場合、それが予想される場合もしくは太い露出血管が視認される場合、救済的に止血鉗子を用います。出血点を鉗子で把持し、Soft 凝固(effect4-6, 80-100W)にて焼灼止血を行います。

② 高周波電源装置：VIO3

使用方法

先端系ナイフに接続して切開・凝固を行います。また止血鉗子に接続して凝固止血を行います。

先端系ナイフの切開：End Cut I モード effect 1 (1-3) duration 2(1-3) interval 1 (1-3)

先端系ナイフの凝固

・従来群：Forced 凝固モード effect 5 (4-6)

・Spray 群：Spray 凝固モード effect 5 (4-7),

止血鉗子の止血：Bipolar-Soft 凝固モード effect 5 (4-6)quick mode

③ 局注針

局注液:局注針から注入する場合には、ヒアルロン酸ナトリウム(ムコアップ;ボストンサイエンティフィック、ケイスマート;オリンパス)もしくはアルギン酸ナトリウム(リフタル K;カイゲンファーマ)のいずれかを用います。ナイフ先端から局注を行う場合には、上記に加えて、生理食塩水もしくはグリセリン溶液を用います

6. 参加予定の被験者数: 150 名の患者様の方へご協力頂くことを予定としています。

7. この臨床試験の実施予定期間とスケジュール

試験参加日:約 1 週間（具体的なスケジュールは下記の表をご参照下さい）

試験に必要な観察及び検査項目:

・臨床研究参加有無を決められた日より以前に確認した胃カメラ所見、自他覚所見(参加決定前 90 日以内であれば他の病院のデータでも構いません)

・下記に示した項目に ESD 当日に確認します。

患者背景（生年月日・性別)・内視鏡所見(部位・サイズ・肉眼型)・
処置時間(全体/粘膜切開/粘膜下層剥離)・術者経験・術者交代・
使用デバイス・ナイフ単独 ESD の完遂の有無・トラクションの有無・
局注液の種類/使用量・切除標本のサイズ・切除標本の状態・内視鏡的遺残の有無・
止血鉗子の使用の有無/使用理由/止血回数/時間、偶発症の有無

・後日、病理結果を診療録で確認します。

眼型・組織型・腫瘍径・水平断端・垂直断端・脈管侵襲・UL の有無・
内視鏡的根治度・粘膜下層の厚さ(切除標本の中央部、健常部における粘膜筋板から標本断端までの距離)、切除検体の熱変性

登録期間：地方独立行政法人北九州市立病院機構治験・臨床研究審査委員会の承認日より 2024 年 3 月 31 日まで（2 年間）
追跡期間：登録日から初回退院日まで
試験期間：許可日～2027 年 3 月 31 日まで（5 年間）

研究全体のスケジュール

検査項目	治療前	同意取得・治療日(1 日目)	治療翌日～退院日
＜両群＞		ESD[1]施行	
胃カメラ	○		
自他覚所見	○		○

1） ESD とは Endoscopic submucosal dissection: 内視鏡的粘膜下層剥離術のことです。

同意取得・検査日(1 日目)の具体的なスケジュール

消化管内視鏡検査で粘膜層に限局する胃上皮性腫瘍が疑われ、生検病理検査で腺腫もしくは腺癌と診断され ESD を受ける患者

↓

同意取得及び登録・ランダム化

↓

患者背景を確認後、割付方法に応じた方法で **ESD**[1]を施行。

↓

処置終了ならびに内視鏡レポートを記載。<br>切除検体を病理部へ提出。

1） ESD とは Endoscopic submucosal dissection: 内視鏡的粘膜下層剥離術のことです。

8. この臨床試験の方法

本臨床研究は、あなたのように、胃上皮性腫瘍（胃腺腫もしくは胃腺癌）と診断され、ESD を行う患者さんを対象に行います。胃の外科手術を受けたことのある方、透析中の方、治療の前後にヘパリンの持続投与が必要となる方、20 歳未満の方、内視鏡検査・治療を受けることができない方、担当医が対象として不適切と判断した患者さん（著しく状態の悪い患者さん）などは、この臨床研究に参加することはできません。文書による同意が得られましたら、患者様さんはコンピューターにより処置の方法を決定いたします。従来法 ESD もしくは Spray-ESD に割付けられます。Spray-ESD による治療のご希望がなく、従来法 ESD を希望される方はお断りいただいても不利益はありません。（「12. この臨床試験に参加しない場合の治療法・検査法について」の項もご参照下さい）。この割付けはコンピューターによって行われ、あなたも担当医もどちらのグループに割付けられるのかは選択できません。つまりあなたは同じ確率でどちらかのグループに割付けられます。いずれか決まった方法で ESD を施行し、病変の摘除を行います。

その後、事前に設定した評価項目を検討することによって、Spray-ESD が本当に役に立っているかどうかを比較検討します。

9. 経済的な負担あるいは謝礼等について

ESD（従来法 ESD および Spray-ESD）は現在の医療保険で胃上皮性腫瘍の患者さんに対して適応が認められており、治療にかかる一切の費用は医療保険制度に添って請求と支払がなされます（この臨床試験は通常の診療の範囲内で行なわれ、用いられる薬剤、検査はあなたの健康保険が適用されます）。ESD で使用する ProKnife，Hemostat-Y もこれらに含まれます。そのため、あなたに特別な費用負担が生じることはありません。

また、本試験に参加いただくことによる謝礼の支払いはありません。

10. この臨床試験による利益と不利益

➤本試験により予期される利益

胃上皮性腫瘍に対する Spray-ESD は本邦における過去のデータからはナイフ単独での ESD 完遂率が上がる（止血鉗子の使用率が下がる）と予想されますが、本当に従来の方法にその効果が上乗せされるかどうかは現時点では不明です。その意味で本試験に参加いただくことによる患者さんご自身の確実な利益は特にありません。

➤本試験により予期される不利益

（1）侵襲等の負担の内容

本研究ではSpray-ESD群で、Spray凝固で粘膜切開・粘膜下層剥離を行います。Spray-ESD群になることにより研究に参加されました患者様への侵襲等の負担が大きくなることはありません。

(2)起こりうる有害事象等について

あなたは、すでに胃上皮性腫瘍と診断され、本臨床研究への参加の有無に関わらず、ESDによる内視鏡治療による病変の摘除が勧められます。ESDは後出血(治療後の出血)、術中穿孔(治療中の穿孔)、遅発性穿孔(治療後の穿孔)などの偶発症があり得ると報告されています。穿孔を来した場合には、それを救済するための治療として外科手術が必要となる可能性があります。本邦で患者さんの数が最も多い報告では、後出血4.4%、術中穿孔2.3%、遅発性穿孔0.4%が報告されています。Spray-ESDにおいてもこれらの偶発症が起こりうる危険性があります。

11. 健康被害が発生した場合の対応と補償について

ESD(従来法ESDおよびSpray-ESD)は現在の医療保険で胃上皮性腫瘍の患者さんに対して適応が認められており、治療にかかる一切の費用は医療保険制度に沿って請求と支払いがなされます。これらの治療によって健康被害が生じた場合の特別な補償の制度はありませんが、病院で誠意をもって治療にあたります。治療は保険を使用した場合の一般診療での対処に準じて行われ、健康保険で定められている自己負担分の費用が発生します。

12. この臨床試験に参加しない場合の治療法・検査法について

この臨床研究に参加されるかどうかはあなたご自身の自由意思によります。これを拒否されても、胃上皮性腫瘍の根治のために必要なESDを通常通り受けることができます。

13. この臨床試験への参加とその撤回について

この臨床試験に参加されるかどうかはあなたご自身の自由意思によります。これを拒否されてもそのことにより不利益を受けることはありません。また同意後治療の開始の有無に関わらずいつでも撤回できます。どちらの場合もその時の病状により専門医として責任をもって最善の治療に当たります。

14. この臨床試験を中止する場合について

あなたがこの臨床試験の中止を希望した場合、病気の悪化や副作用のために担当医が試験を中止した方がよいと判断した場合は試験を中止します。試験中止後も、その後の対応について担当医が誠意をもって相談に応じます。

15. この臨床試験に関する情報の入手及び閲覧について

この試験に関して、参加の継続についてあなたのご意思に影響を与える可能性のある情報が得られた場合にはすみやかにお伝えします。また、この試験に関する資料をご覧になり

たい場合は、可能な範囲で閲覧いただけるように手続きをいたしますのでお申し出下さい。

16. 公開データベース登録について

本試験の概要（試験の名称、目的、方法、実施体制、試験対象者の選定方針等）は、国立大学附属病院長会議の公開データベース「UMIN」に登録します。試験参加者個人が特定される情報は公開されません。

17. 個人情報保護、試料・情報の保管及び廃棄の方法について

この臨床試験の結果は、学会発表や論文での報告、特許などに使用しますが、あなたご自身のプライバシーに関する秘密は全て厳守します。氏名などの個人を特定する情報は、報告に当たって一切使用しません。本試験で得られた試料・情報は、「人体から取得された試料及び情報等の保管に関する標準業務手順書」に従って、少なくとも試験終了後 5 年間厳重に保管の上、適正に廃棄いたします。当院の個人情報管理責任者は、北九州市立医療センター・消化器内科・主任部長・隅田頼信が行います。なお、この臨床試験が正しく行われていて秘密が守られることを前提として、モニタリングや監査、倫理審査委員会関係者などが、必要な範囲内で、この試験に参加していただいている皆さまの 試料・情報を閲覧する場合があります。

本試験で得られた試料・情報は、「人体から取得された試料及び情報等の保管に関する標準業務手順書」に従って厳重に保管いたします。本試験で得られたデータを別の試験に2次利用する場合は、改めてその試験計画を倫理審査委員会において審査し、承認を受けた上で利用します。この場合も、あなたの実名を出すようなことは一切ありません。あなたの病状や名前などに関する情報を含めプライバシーは厳重に守ります。なお、この臨床試験が正しく行われていて秘密が守られることを前提として、モニタリングや監査、倫理審査委員会関係者などが、必要な範囲内で、この試験に参加していただいている皆さまの 試料・情報を閲覧する場合があります。

18. この臨床試験の資金と利益相反について

当院では、より優れた医療を社会に提供するために積極的に臨床試験を推進しています。そのための資金は、公的資金以外に企業や財団からの寄付や契約でまかなわれることもあります。現代社会では医学試験の発展にとって、企業との連携は必要不可欠なものとなっており、国や大学も健全な産学連携を推奨しています。

一方で、産学連携を進めた場合、臨床試験が企業の利益のためになされるのではないかとか、試験についての説明が公正に行われないのではないかといった疑問が生じることがあります。このような状態を「利益相反」―患者さんの利益と試験者や企業の利益が相反（衝突）している状態―と呼びます。患者さんの利益が最優先されるべきであることは当然のことですが、臨床試験においてはその判断がきわめて難しくなっています。

そのような問題に対応して、臨床試験に参加する予定の人々には、その研究の資金源も含めて、十分な説明がなされなければならないことが国際的なルールとして定められています。これに対応して、当院においても利益相反の管理に関する規定が定められています。これらに基づいて、以下のように対応することとしております。

本試験にかかわる研究代表者である北九州市立医療センターの隅田頼信は、本治験で使用する試験機器を製造販売する企業及び関連企業から寄付や規定額(50 万円)を超える講演料などの支援を受けていません。これらの情報を提供した上で、利益相反状態がないことを含め臨床試験実施計画は倫理委員会で審議され、承認されました。利益相反の詳細についてもっと詳しく知りたい場合は、北九州市立病院機構臨床研究推進センター(電話:093-533-5666)までお問い合わせ下さい。

19. 特許権等について

この試験の結果として特許権などが生じる可能性がありますが、その権利は研究代表機関である北九州市立医療センターおよび九州大学に属し、あなたには属しません。また、その特許権などを元にして経済的利益が生じる可能性がありますが、これについてもあなたには権利はありません。

20. お守りいただきたいこと

この試験に参加していただける場合には、以下のことをお守りください。

・試験参加期間中は、担当医の指示に従ってください。

・他の病院を受診したい場合もしくは受診の予定がある場合、市販のお薬を使用したい場合は、必ず事前に担当医に相談してください。

21. この臨床試験の実施体制と連絡先(相談窓口)

研究代表者

北九州市立医療センター・消化器内科・主任部長・隅田　頼信

共同研究機関　研究責任者及び研究分担者等

研究責任者　北九州市立医療センター・消化器内科・主任部長・隅田　頼信
九州大学大学院医学研究院・病態制御内科学・助教・蓑田　洋介
福岡中央病院・消化器内科・部長・河邊　毅
済生会二日市病院・消化器内科・部長・岩佐　勉

研究分担者　北九州市立医療センター・消化器内科・副院長・秋穂　裕唯
北九州市立医療センター・消化器内科・部長・國木　康久
北九州市立医療センター・消化器内科・部長・福田　慎一郎
北九州市立医療センター・消化器内科・部長・塩月　一生
北九州市立医療センター・消化器内科・副部長・前原　浩亮
北九州市立医療センター・消化器内科・副部長・大角　真央
北九州市立医療センター・消化器内科・専修医・近藤　悠樹
九州大学大学院医学研究院・病態制御内科学・助教・荻野　治栄
九州大学大学院医学研究院・病態制御内科学・大学院・江崎　充
九州大学大学院医学研究院・病態制御内科学・大学院・畑　佳孝
九州大学大学院医学研究院・病態制御内科学・大学院・和田　将史

九州大学大学院医学研究院・病態制御内科学・大学院・水流　大尭
九州大学大学院医学研究院・病態制御内科学・大学院・木村　勇祐
九州大学大学院医学研究院・病態制御内科学・医員・鈴木　祐輔
九州大学大学院医学研究院・病態制御内科学・医員・稲田　泰亮
福岡中央病院・消化器内科・医長・中村　典資
福岡中央病院・消化器内科・医長・北川　祐介
済生会二日市病院・消化器内科・部長・志賀　典子
済生会二日市病院・消化器内科・部長・西岡　慧
済生会二日市病院・消化器内科・医長・丸山　薫

この研究のことで何かわからないことや心配なことがありましたら、いつでも、ここに記載されている医師または相談窓口にお尋ねください。

研究事務局
北九州市立医療センター・消化器内科・主任部長　隅田　頼信
電話番号 093-541-1831 内線 6921

同　意　書

北九州市立医療センター　院長　殿

臨床研究課題名
「胃上皮性腫瘍に対する従来法 ESD および Spray-ESD の無作為比較試験」

1. 臨床試験について
2. あなたの病気について
3. あなたの病気に対する治療法について
4. この臨床試験の目的、背景、意義
5. この臨床試験で使用する薬剤・医療機器について
6. 参加予定の被験者数
7. この臨床試験の実施予定期間とスケジュール
8. この臨床試験の方法
9. 経済的な負担あるいは謝礼等について
10. この臨床試験による利益と不利益
11. 健康被害が発生した場合の対応と補償について
12. この臨床試験に参加しない場合の治療法・検査法について
13. この臨床試験への参加とその撤回について
14. この臨床試験を中止する場合について
15. この臨床試験に関する情報の入手及び閲覧について
16. 公開データベース登録について
17. 個人情報保護、試料・情報の保管及び廃棄の方法について
18. この臨床試験の資金と利益相反について
19. 特許権等について
20. お守りいただきたいこと
21. この臨床試験の実施体制と連絡先（相談窓口）
22. その他特記事項

【患者さんの署名欄】

私はこの研究に参加するにあたり、上記に関する説明を十分理解した上で、臨床試験に参加することに同意します。また、この同意はいつでも撤回できることを確認しています。

同意日：　　　　年　　　月　　　日

患者さん氏名（自署）＿＿＿＿＿＿＿＿＿＿＿＿＿＿＿＿＿＿＿＿

【代諾者の署名欄】＊代諾者ありの場合は欄を設けて下さい（ない場合は削除して下さい）。

私は＿＿＿＿＿＿＿＿＿＿＿＿＿さんが、この研究に参加するにあたり、上記に関する説明を十分理解した上で、臨床試験に参加することに同意します。また、この同意はいつでも撤回できることを確認しています。

同意日：　　　　年　　　月　　　日

代諾者氏名（自署）＿＿＿＿＿＿＿＿＿＿＿＿＿＿＿＿＿＿　続柄＿＿＿＿＿＿

【研究責任医師又は分担医師の署名欄】

私は、上記の患者さんに本研究について十分に説明しました。

説明日：　　　　年　　　月　　　日

説明者氏名（自署）＿＿＿＿＿＿＿＿＿＿＿＿＿＿＿＿＿＿＿＿
